# Medical doctors in healthcare leadership: theoretical and practical challenges

**DOI:** 10.1186/s12913-016-1392-8

**Published:** 2016-05-24

**Authors:** Jean-Louis Denis, Nicolette van Gestel

**Affiliations:** École nationale d’administration publique (ENAP), Montreal, QC H2T 3E5 Canada; TIAS School for Business & Society, Tilburg University, Warandelaan 2, Tilburg, 5037 AB The Netherlands

**Keywords:** Medical engagement, Clinical leadership, Health system improvement

## Abstract

**Background:**

While healthcare systems vary in their structure and available resources, it is widely recognized that medical doctors play a key role in their adaptation and performance. In this article, we examine recent government and organizational policies in two different health systems that aim to develop clinical leadership among the medical profession. Clinical leadership refers to the engagement and guiding role of physicians in health system improvement. Three dimensions are defined to conduct our analysis of engaging medical doctors in healthcare leadership: the position and status of medical doctors within the system; the broader institutional context of governmental and organizational policies to engage medical doctors in clinical leadership roles; and the main factors that may facilitate or limit achievements.

**Methods:**

Our aim in this study is exploratory. We selected two contrasting cases according to their level of institutional pluralism: one national health insurance system, Canada, and one etatist social insurance system, the Netherlands. We documented the institutional dynamics of medical doctors’ engagement and leadership through secondary sources, such as government websites, key policy reports, and scholarly literature on health policies in both countries.

**Results:**

Initiatives across Canadian provinces signal that the medical profession and governments search for alternatives to involve doctors in health system improvement beyond the limitations imposed by their fundamental social contract and formal labour relations. These initiatives suggest an emerging trend toward more joint collaboration between governments and medical associations. In the Dutch system, organizational and legal attempts for integration over the past decades do not yet fit well with the ideas and interests of medical doctors. The engagement of medical doctors requires additional initiatives that are closer to their professional values and interests and that depart from an overly focus on top down performance indicators and competition.

**Conclusions:**

Different institutional contexts have different policy experiences regarding the engagement and leadership of medical doctors but seem to face similar policy challenges. Achieving alignment between soft (trust, collaboration) and hard (financial incentives) levers may require facilitative conditions at the level of the health system, like clarity and stability of broad policy orientations and openness to local experimentation.

## Background

While healthcare systems vary in their structure and available resources, it is widely recognized that medical doctors play a key role in the adaptation and performance of these systems [1, 2]. Physicians have a unique influence on the utilization of healthcare resources by prescribing treatments and drugs. They can play various formal and informal roles that help creating a rich environment for improved practices and ultimately increase the performance of healthcare organizations [3, 4]. Studies on health system performance and clinical governance emphasize the importance of strong clinical leadership to drive improvement efforts and initiatives [5, 6]. Hospital performance is increasingly associated with medical specialists taking up tasks beyond direct patient care and develop their co-operation with executive boards [7, 8]. Leadership and engagement of other professionals are also crucial for health system improvement; yet, the unique status and influence of the medical profession may require a specific focus of attention.

In this article, we examine recent government and organizational policies that aim to develop, implement, sustain and scale-up clinical leadership among the medical profession. Clinical leadership refers to the engagement and guiding role of physicians in health system improvement. This role goes further than their involvement in formal leadership positions. It refers to an active role of doctors in activities for healthcare improvement that goes beyond their immediate clinical duties and responsibilities in delivering care to patients [9]. Spurgeon and colleagues (2008) suggested that these activities can include the participation of doctors in managing risks and quality; the evaluation of programs or technologies at organizational or system levels; the involvement in strategic committees that influence the development of the organization; or the involvement of physicians in executive roles [10].

Those roles and expectations regarding professionals and more specifically medical doctors have been designated as “professional-managerial hybrids” or clinical leaders [11, 12]. Clinical leadership thus incorporates a variety of roles and resources that help front-lines clinicians to introduce new ways of working and to redesign care for improvements [4]. It is expected that clinical leaders will influence their peers through their professional knowledge and skills in promoting improvement of care within the context of available resources. They will also collaborate with managers in developing organizational strategies that are aligned with quality improvement [13].

While studies have shown benefits in the development of clinical leadership where clinical expertise is combined with other capacities; the materialisation and broad-scale diffusion of clinical leadership for improvement within healthcare systems is not without challenges [2, 12]. Professional power may resist attempts by clinical leaders to reframe the context of work and the relationships between organizations and professions. The position of a professional elite that gains power and control over their peers in exchange of the protection of professional autonomy - the restratification thesis of Friedson (1984) - may be contested [14, 15]. Because of those potential challenges, governments and organizational policies search for strategies to mobilise a broader professional base to improve care such as collaborative quality improvement initiatives [16]. A large-scale development of clinical leaders for improvement within healthcare systems needs the support of institutional conditions, such as career perspectives and the development of skills and capacities to engage in mediating roles between organizations and the professions [11, 17]. Studies of organized professionalism and hybrid roles [13, 18] have suggested the importance of institutional factors that promote or impede the emergence of such leadership roles. Studies of medical doctors in management have focused on the incorporation of new logics or imperatives in clinical practices (for example accounting in medicine in Finland [19]), impact of quasi-market in NHS-England on the emergence of medical-managers roles [20], and more recently, forms of identities in hybrid roles among medical managers [12]. While these works are very informative on the emergence and characteristics of hybrid roles among the medical professions, they do not take a system-wide context and health policy perspective on the strategies used to engage medical doctors in hybrid roles and more broadly in improvement efforts. In this paper we look at recent developments in the institutional context of medical doctors in two very distinct healthcare systems. Our empirical analysis is focused on how government and organizational policies may (or may not) drive and shape the emergence of clinical leaders for improvement.

The paper is structured as follows. We first review literature on the challenges in engaging medical doctors in system and organizational leadership roles. Then we investigate how two different healthcare systems shape or limit the opportunities for physician engagement and leadership. We use the illustrative cases of Canada and the Netherlands to analyze recent experiences of medical doctors’ leadership and engagement. Based on key studies in the field, we discuss strategies to better engage medical doctors and develop their leadership for health system improvement.

## Challenges in reconciling professional and organizational logics in healthcare

Professions have incarnated an idealized form of expertise in contemporary societies, with an ability to skillfully apply complex knowledge to the resolution of problems. The medical profession still represents an ideal-typical form of profession where competent individuals provide highly valued services in unselfish devotion [20] and where professional autonomy is exercised in the context of accountability to patients and peers [21]. Sociological studies of the medical profession have emphasized the professionals’ capacity to preserve monopoly over specialized knowledge and to create boundaries that protect the status and roles of doctors in society [22, 23].

Initiatives that aim at involving medical doctors in roles that go beyond the delivery of clinical services will necessarily imply a combination of their professional expertise with other forms of knowledge, and the application of this expertise to more collective problems and issues [9]. This is also recognized in the notion of “organized professionalism” where medical practice is perceived as increasingly embedded in a broader organizational context, due to a set of political, economic and social forces and contingencies that challenges the traditional representation of professional work and independence [13].

Several studies have focused on the theme of the integration and alignment of the medical profession within US healthcare organizations [24–26]. These researchers examined organizational models and strategies that promote a stronger connection between the medical profession and healthcare organizations and systems through various structural and incentives arrangements. One of the key insights gained from these works is that structural and economic integration of medical doctors within organizations are not sufficient to enable genuine engagement and leadership for improvement.

Other studies have paid attention to the accommodation of the managerial and professional logic in knowledge-based and professional organizations [27]. Medical doctors and other healthcare professionals face increasing pressures to work within organizations and to become more involved in formal organizational settings [28, 29]. Evidence regarding the impact of organizational context on the professional status and practices [27, 30, 31] indicates however that the medical profession has adapted quite well to practice in more formal organizational contexts. Such adaptation can reflect a new balance between professional norms and organizational demand for accountability while revealing at the same time the ability of some professions to operate a kind of organizational closure [32] or to restructure their work in favour of more collaborative forms of work to simultaneously achieve professional and organizational goals [30].

Although a process of accommodation between the medical profession and organizations has been observed in these studies, they do not provide much insight into how to convert such accommodation into resources for professional renewal and health system improvement. Analysis by Waring [15], Schott & al. [18] and Muzio et al. [32] suggest that the roles of professionals and their autonomy are in a constant flux where various forms of professionalism - occupational, organizational, hybrid - interact to create a kind of situated professionalism, influenced by broader changes in the institutional context. These studies stay relatively silent on how increased social demands for more accountable professions influence the development, transformation and use of clinical capacities for leadership. More specifically, we lack knowledge on how contingencies at the level of (healthcare) systems and organizations induce a reframing of professional engagement and clinical leadership. Attempts to incorporate physicians into organizational structures are common now, but appear insufficient by itself for engaging doctors in the redesign and regulation of health care systems [4]. The political context and government policies, in particular policy capacity and coherence [33], can also influence the propensity of doctors to engage in healthcare improvement and in new regulatory roles and functions.

The transition of (some) medical doctors to leadership positions in healthcare organizations and systems is thus associated with several conceptual and empirical challenges. One challenging issue is about the capacity of professionals to invest in more collective levels of application of professional knowledge and expertise while maintaining their specificity and identity in more collective and distributed forms of leadership [34]. Specifically, the perceived needs for increased regulation to produce high quality and safety of care [35] may support the emergence of a new professional elite [14, 15] and a new science of healthcare improvement that may contradict with the broader movement of engaging physicians and other front-line professionals in large-scale improvement [36]. Also, the development of new regulatory tools for making clinical work more manageable and visible will necessarily impact on the relationships of medical doctors with their work context and practice settings [2].

Based on our literature review, we clearly reveal three dimensions that are related to the theme of engaging medical doctors in healthcare leadership: the position and status of medical doctors within the system; the broader institutional context of governmental and organizational policies to engage medical doctors in clinical leadership roles; and the main factors that may facilitate or limit achievements. We will analyze these three dimensions in the public healthcare systems of Canada and the Netherlands, and evaluate recent initiatives to engage medical doctors and develop their leadership for health system improvement. In the next section, we first explain our methodology.

## Methodology

Our aim in this study is exploratory and based on the assumption that the broader institutional context of government an organizational policies influences the propensity of medical doctors to move beyond their traditional role as the patient’s agent to develop clinical leadership. We selected two cases for the current study with different institutional characteristics: the Canadian and the Dutch healthcare systems. These systems represent two contrasting cases [37] regarding the influence of broad institutional and health system context on the development of clinical leadership for improvement. The two countries have been categorized in a recent health system typology as a National health insurance system (Canada) and an etatist social health insurance system (the Netherlands) [38].

The two cases vary on key organizing dimensions: financing for Canada is through fiscal resources, regulation is operated by the State and the system relies on private or not-for-profit providers that are covered by public money for medical and hospitals services. In the Dutch case, regulation is also operated by the State, but the role of private providers (like insurers companies) in the governance and finance of healthcare has been significantly increased since the reform in 2006. A key feature of the Canadian healthcare system is the strong autonomy of the medical profession with a focus on negotiating provision and compensation directly between the State (provinces) and medical associations [39]. In the Dutch case, the system is much more diverse, and leaves more space for various arrangements or levels of integration between the medical profession and delivery organizations. The Dutch system is by its pluralistic nature, as compared to Canada, apparently less centralized, with probably more space for organizational-professional initiatives than the Canadian one.

Based on the work of Tuohy [40] on hybridization process in mature healthcare systems we argue that some systems leave more space for professional entrepreneurship and are more receptive for reforms to improve and adapt the healthcare system (for example the Netherlands), while some other systems will be more characterized by inertia (e.g. Canada) [39]. Consequently, systems may offer more or less opportunities for the involvement of doctors in clinical leadership at both the organizational and system levels, for example in professional-managerial hybrid roles [12] or in broader improvement process such as collaborative quality improvement. Aligned with the exploratory scope of this article, we have documented the institutional dynamics of medical doctors’ engagement and leadership in both jurisdictions through secondary sources, such as websites of governments in each jurisdiction, identification of key policy reports, and scholarly literature on health policies in both countries.

## Transforming the role of the medical profession: the Canadian case

In Canada, provinces and territories are responsible for the management, organization and to a large part for the financing of their healthcare system. Each province and territory develops its healthcare system within the broader context of the *Canada Health Act* (CHA or the Act, adopted in 1984). The Act is Canada’s federal legislation for publicly funded health care insurance and sets out the primary objective of Canadian health care policy [41]. Overall, the costs of the healthcare system in Canada are estimated at 11.2 % as a share of GDP for an average of 9.3 % for OECD countries [42]. Cost is considered an important policy issue while for provinces the cost of healthcare represents more than 42 % of the expenditure in public programs. The Commonwealth Fund ranks the Canadian healthcare system poorly to some dimensions of quality, patient experience and access [43]. Pressures are important to improve the functioning of these systems and the care that is delivered.

### The position of medical doctors in Canada

The basic social contract of Canadian medicine is one of autonomy, professional entrepreneurship and arms-length relationships with health systems and governments. At the time of the creation of the public healthcare systems in various provinces, it was agreed that physicians would not be considered as employees of public healthcare organizations. They mostly maintained a capacity to operate as autonomous agents in the system, paid by an independent public agency or third-party payer [39]. Physician unions (Quebec) or professional associations negotiate directly their status and practice conditions through labour agreements with provincial and territorial governments. Concretely, medical doctors obtained the privileged right to practice in hospitals/public healthcare organizations, through the boards of healthcare organizations and local medical councils, and medical doctors are more or less regulated by their peers through various organizational arrangements and professional colleges depending on the provinces or territories.

This type of social contract between the medical profession and the health care systems and more largely with the society, has been overall very beneficial for the profession. They still benefit from a lot of autonomy in regard of their location of practice and development of their professional career; they have a very high social status, and they are very well paid. The income of physicians relative to average wage in Canada is estimated at 4.7 for specialists and 3.1 for generalists [43]. Recent statistics from OECD (2014) shows that medical workforce has grown since 2000 but the number of physicians in Canada (2.5 doctors per 1000 population) still remained below the OECD average of 3.2 [44]. The growing number of physicians may put more pressures on costs and consequently push governments in a better position to develop initiatives to better engage medical doctors in health systems improvement.

Overall, recent analysis [39] suggests that one of the key obstacles to reform healthcare in Canada is related to the ability of the medical profession as an organized body to defend their interests and resist changes that are perceived against these interests. Those authors used the term “*paradigm freeze*” to qualify the inertia within the Canadian healthcare system that emanated, at least partly, from the fundamental social contract between the medical profession and the state that somewhat consecrated a situation of arms-length relationship between doctors and governments. We will now look at strategies that aim at transcending these structural limitations in order to better involve medical doctors in broad system improvement.

### Initiatives to engage medical doctors in leadership for healthcare improvements

Within the broader context just described, there are emerging initiatives across Canada to better engage medical doctors and develop their leadership for health system improvement. We will describe here some of these initiatives in various provinces and assess how they depart from a more regulatory approach and support the development of the commitment of the profession for broader health system goals [45]. The purpose of this case description is not to provide an exhaustive account but to briefly account for illustrative purposes of some trends that are informative on how clinical leadership of medical doctors develop within and depart from the situation of *“paradigm freeze”* as we described earlier.

Our analysis of these initiatives reveals two emerging trends in the Canadian health policy scene with regards to doctors and the healthcare system: the emergence of collaborative work for improvement between the medical profession and governments and a growing emphasis on accountability relations and performance management in shaping the relation between the profession and public authorities. Those trends are not mutually exclusive, they can compete for legitimacy which may create tensions in the relation between medical doctors and government. We will now describe some initiatives in different provinces to illustrate the dynamic that surrounded efforts to develop clinical leadership for health system improvement among the medical profession.

In *British Columbia*, the third larger province in Canada, the Shared Care Committee is created in 2006 [46]. The committee is a joint initiative between the British Columbia Medical Association (BCMA) and the British Columbia Ministry of Health. The purpose of the committee is to support initiatives among the medical profession to improve care in the system. Government funding is provided to support such initiatives For example, in 2013, $8 million has been provided to support 21 medical-led initiatives through the SCC. These initiatives appear to be based on two principles: they leave a lot of flexibility to local and regional initiatives in the design of the different projects and they are essentially collaborative where the professions and more specifically the medical profession is a key driving force.

These initiatives can be considered as a political trend toward the adoption of a less adversarial approach in the relation between the medical approach and government [47]. It also suggests that a medical leadership for improvement agenda becomes integrated within the discussions between the state and the medical association and consequently broaden the negotiation space beyond the working conditions of professionals.

*Saskatchewan*, a province from central Canada, provides illustration of a different approach with the launch in 2008 of a large-scale improvement program - the Accelerating Excellence program [48] - to develop quality improvement initiatives and capacities across their healthcare system. This program is strongly driven by the central government and the diffusion of the Lean approach has been privileged as the main driver of improvement: “Saskatchewan is the first jurisdiction in Canada to apply Lean processes across its entire health system. More than 1000 Lean projects have been launched in Saskatchewan health regions and within the Ministry of Health and Saskatchewan Cancer Agency” (http://www.saskatchewan.ca/government/health-care-administration-and-provider-resources/saskatchewan-health-initiatives/lean). It is within this broader provincial effort to improve the system that medical association and professional college in collaboration with the government the Champions for Quality Improvement initiative to support the adhesion of doctors to the Accelerating Excellence program [47]. Policy trends in favour of health system improvement provide opportunities for the development of clinical leadership among the medical professions. The level at which the involvement of doctors in the implementation of the quality agenda will culminate in a large-scale development of medical leadership for improvement still an open question. As in British-Columbia, policy-makers and professional association have agreed that to involve medical doctors beyond their immediate clinical duties specific strategies need to be developed taking into account their singular position and status in the system.

One of the driving forces across Canada behind a greater co-optation of the medical profession in improvement initiatives are pressures from governments for increased accountability and performance. For example, *Ontario*, the largest province in Canada, has promulgated the Excellent Care for All Act in 2010. A set of initiatives have cascaded down from this act including new funding and incentive mechanisms and approaches that place distinct emphasis on quality and on the role of medical doctors in achieving improvements in healthcare [47]. In addition, the Act introduced Health Quality Ontario, an agency responsible for the provincial emphasis on quality. Medical compensation has also been increasingly tied to evidence based recommendations and performance targets, with a major emphasis on primary care [49]. Ontario, somewhat similar to the situation in Saskatchewan, illustrates a situation where a strong policy-drive from the provincial government set up a new context to approach the role of medical doctors in improvement. An important role is attributed here to the management of performance and to the use of incentives to stimulate involvement in quality improvement.

*Quebec*, the second largest province of Canada, has promoted the engagement and the leadership of medical doctors for health system improvement through time with various structural arrangements like the creation of medical advisory or planning bodies at the central government and regional levels and various executive positions for medical doctors within the health care system. A current healthcare reform combined elements of massive restructuring across the system (effective April 1, 2015), budget rationalization and a new policy to set productivity targets for primary care physicians. Medical doctors’ union engaged in intense negotiations with the government to postpone the application of the new bill in exchange of a guarantee to meet productivity targets [50].

These major policy initiatives reflect the importance played by the regime of labour relations and the social contract between the medical profession and the State in shaping the involvement of doctors in improvement initiatives. Development of clinical leadership for improvement among medical doctors is also strongly influenced by the broader context of “big-bang” reform in the Quebec healthcare system. Compare to the three others provinces, the implementation of large-scale restructuring may have left less space for the development of collaborative work for improvement between medical doctors and the government. While in all provinces the institutional arrangements associated with the labour negotiation regimes influence the framing of expectations and the roles taken by the medical profession in improvement initiatives, the history of broad structural reforms in Quebec suggests that it has somewhat made more challenging to develop a more collaborative policy agenda.

### Facilitative and limiting factors within the Canadian cases

These initiatives across Canadian provinces signal that the medical profession and/or governments search for alternatives to involve doctors in improvement initiatives beyond the limitations imposed by their fundamental social contract and formal labour relations regimes. While the examples we discussed kept the attributes of a top-down policy process (Quebec, and to a lesser degree Saskatchewan and Ontario); the shared objective of improving patient care and the pressures for increased performance and accountability seems to favour the development of a more collaborative approach around specific policy initiatives. Joint improvement work and more affirmative performance management can be a way to transcend a situation of “paradigm freeze” that have characterized healthcare reforms in Canada [39].

One may retain from our analysis of the Canadian cases that the ability to engage the medical profession in large-scale improvement cannot be understood without paying attention to the broader health system policies and governance context. Strategies to engage doctors are, at least partly, conditioned by the labour regime and by the policy-drive of governments to support large-scale improvement. Collaborative work between medical doctors and the state is still an emerging phenomenon. It is too early to assess the implications of these policies and initiatives for the evolution of medical professionalism including for the constitution of a new medical elite that will carry on the improvement agenda. In addition, the capacities of medical doctors involved in improvement work to impose themselves or to mobilise rank and file doctors at a sufficiently large-scale are still an open question. It is also too early to assess the performance agenda within the regulation of the medical profession and its influence on their behaviors as providers of care.

## Engaging medical doctors in healthcare leadership: the Dutch case

In the Netherlands, the national government is responsible for regulating the healthcare system and setting main strategic priorities. Hospitals and primary healthcare services develop their management and care activities within the context of the Dutch Health Insurance Law (the ‘Zorgverzekeringswet’, Zvw), introduced on 1 January 2006. The Health Insurance Law is a mandatory ‘basic insurance’ that covers common medical care and medicines. For long-term nursing and care, there is another statutory form of insurance, the Long-term Care Act (‘Wet Langdurige Zorg’, WLZ), introduced on 1 January 2015. Dutch residents are automatically insured by the government for WLZ, but have to choose and pay individually for their basic healthcare insurance. Health insurers have to offer a universal package for everyone, regardless of age or health conditions, but may compete for price. Contrary to many other European systems, the Dutch government is responsible for the accessibility, quality and ultimate costs of the healthcare system, but not in charge of its management [8]. Private health insurers have a pivotal role since the Health Insurance Law in a system of managed competition. Although private (mainly not-for-profit) organizations play a main role in executing the Dutch healthcare system, 85 – 90 % of the health care sector is collectively financed through compulsory contributions and taxes.

Overall costs of the healthcare system in the Netherlands are estimated at 11.8 % of Dutch GDP in 2012 [44], which is around the level in Canada and above the OECD average. Cost containment is one of the most important issues in the negotiations between the Dutch national government, health insurers and healthcare providers, such as hospitals. In comparing healthcare systems in Europe on indicators such as patient rights and information, accessibility, prevention and outcomes, the annual Euro health consumer index (EHCI) found in 2014 that the Netherlands maintained its top position from the past five years. The Commonwealth Fund also shows that the Dutch system has generally high scores on performance; however, several aspects such as accessibility, prevention, and the varying quality and costs of healthcare providers show a clear room for improvement [43].

### The position of medical doctors in the Netherlands

Traditionally, medical doctors have a strong position in the Netherlands. This is due to their high professional status, but also to relatively low numbers, compared to many other European countries. For example, Germany and Denmark have twice as much medical specialists per 1,000 inhabitants than the Netherlands [51]. Within healthcare organizations, and in particular hospitals, the position of medical specialists is distinct from most other European countries. While nearly all physicians in hospitals in France, England, Denmark and Germany are employees; in the Netherlands only 40 % of the approximately 21,000 medical doctors in hospitals have an employee status. The other 60 % is entrepreneur and allied to the hospital with a special management agreement [51]. In 2012, the Dutch government decided for a revenue ceiling for self-employed doctors, which will be implemented gradually over the next years. Although the position of medical doctors with an entrepreneurial status in the Netherlands is still strong, there are (ongoing) political pressures for more standardization and integration of medical doctors in hospital governance.

### Initiatives to engage medical doctors in leadership for healthcare improvements

Over the past decades, there have been main attempts in the Netherlands to integrate medical specialists in hospital governance [8, 52]. Already in the 1980s, medical specialists were supposed to play a more crucial role by ‘getting involved in management tasks on a clinical- (organising care) and organisational-level (hospital as a whole)’ ([8] p. 325). Such initiatives to engage medical doctors in leadership roles beyond immediate patient care were however accompanied by governmental policies to restrict their independent status as non-employees and related (higher) incomes [52]. The initiatives to involve doctors in hospital management while simultaneously limiting their revenues led to tensions and barriers between hospital boards and medical specialists in developing common policies for healthcare improvements.

In a context of growing concerns about rising health care expenditures, in the mid-1990s, medical specialists and executive boards of hospitals started to take up joint responsibility for setting up and launching a strategic direction for the hospital; the so-called Integrated Medical Specialist Organisation model [52]. In these initiatives, medical specialists were expected to take more responsibility for organisational tasks and development, ‘which meant that medical work no longer contained medical activities alone, but consisted of inter-disciplinary managerial activities ([8] p.325). At the level of the broader healthcare system, major national healthcare organizations provided joint agreements for healthcare improvement. For example, the joint Health Care Sector Organisations (hospitals included) took the initiative to establish the Care-Wide Governance Code for good management and supervision [53]. This Governance Code is accepted and applied by every healthcare organisation in the Netherlands; it defines among others the responsibilities of the executive board, having the final responsibility for managing the healthcare organisation and its risks, and for ensuring that all medical specialists, either employees or entrepreneurs, fulfil their responsibilities. Given increasing pressures for cost containment, and growing concerns for healthcare quality, the various stakeholders at different levels in the healthcare system thus took initiatives to develop closer ties and common views between hospital management and medical doctors.

Over time, the theme of governance, quality and safety received ever more attention in healthcare, with an increasingly prominent role for professionals, such as medical specialists. In 2009, the Council for Public Health and Care delivered its opinion that healthcare governance cannot function without professionals being held accountable for their actions in the report “Governance and Quality of Care” (2009) [54]. This report was supplemented with an advisory letter on the “Relationship between the medical specialist and the hospital in the light of the quality of care.” (2010) [55]. Other influential organizations also argued for improving the healthcare system on quality indicators. For example, the Netherlands Court of Audit produced a critical report on the Evaluation of the Quality of Care Institutions (2009) stating that quality standards are insufficiently and should be better monitored [56]. And the Healthcare Inspectorate expressed its critical vision in the report ‘Beyond permissiveness. Control and monitoring of quality and safety’ (2009) [57]. These critical views were incorporated in a new regulatory framework of the IGZ-Toezichtkader [58] for supervision of healthcare systems’ quality and safety. The Order of Medical Specialists, being the largest professional association of medical doctors, published a ‘Quality Framework’ (2010) about the relationship between medical specialists and boards. A most critical issue in this code of conduct is that medical specialists and executive boards should work together to guarantee quality and improving specialist medical care. We thus perceive a growing emphasis on the necessity for engaging medical doctors in integrated efforts for improving the Dutch healthcare system.

In the Netherlands this has resulted in a recent government reform (2015) for a new financial structure and incentives for collaboration between hospital boards and medical specialists. From 1 January 2015 onwards, an integral tariff has been introduced for hospital medical care and two budgets which were formerly distinct and separated are now allied: the budget of the hospital and the fee budget for medical specialists. The government reform aims to encourage hospitals and medical specialists for a more intensive and long term collaboration, to ensure that the hospital is sufficiently prepared for the future in terms of care functions and costs. The government reform is meant to stronger unite the goals of hospitals and (in particular self-employed) medical doctors, to let them develop jointly the strategy and future of hospital care.

The introduction of bundled payment implies that the hospitals and medical doctors should discuss together the hospitals’ policy and have to negotiate the fees of medical specialists. As a result of the recent reform, the hospitals and medical doctors are searching for a new model for management and organization. Until 2015, most self-employed medical doctors in the Netherlands were organized in so called partnerships: a group of specialists, who usually share the same specialty and provide care to a particular patient group. Given the 2015 government reform, this partnership form is currently under discussion. Basically, there are three alternatives [8]. First, the Salaried Model where the self-employed medical doctors become employed at the hospital as employees. A second model is the Cooperation Model, where medical specialists organize themselves in their own organizations (Medical Specialist Companies) that can conclude an agreement on collaboration with the hospital. A third option is the Participation Model where medical doctors become co-owners of the hospital. So far, in most hospitals in the Netherlands, medical doctors have chosen for the second model and started Medical Specialist Companies (MSCs) on a cooperative basis. Much attention has been spend and is still focused on the new structure for management and organization, which does not necessarily imply increasing engagement of medical doctors in activities beyond direct patient care or formal leadership roles.

### Facilitative and limiting factors within the Dutch case

Within the broader healthcare system in the Netherlands, the position of the medical specialist in relation to the hospital has been developed from coexistence to dialogue and formal models for integration in the past decades [59]. Several system reforms, in particular the market-based reforms with the Health Insurance Law in 2006 and a new funding system for reimbursing medical treatment (DTCs), have created a strong mutual dependence between organizations and professions in the healthcare field for improving and controlling the quality of care, the volume (production) and cost/benefit ratios [59]. Mutual dependence between healthcare organizations and medical doctors is also evident in the internal organization of hospitals wherein hospital units are increasingly headed by a medical specialist and a manager of business who are together fully responsible for quality, production, personnel and finances. The new regulations, financial incentives and organizational changes are aimed to facilitate health systems’ progress. They may create the conditions for medical doctors to become stronger involved in roles for improvement that go beyond their direct responsibilities for patient care and collaborating with peers.

Limitations of the initiatives so far seem to be found in the emphasis on structure, finance and organization rather than on process, communication, and professional values. Although the 2015 governmental reform aimed for integrating the medical specialists in hospitals and created a better basis for a collaborative effort in developing the hospital strategy and improving healthcare; it seems as if it does not guarantee more collaboration yet and even may work out in opposite direction. For example, the new Medical Specialist Companies have increased the collective autonomy of self-employed medical doctors, which result in a stronger position for negotiation with the hospital boards but does not necessarily lead to more engagement of medical specialists in leadership roles that go beyond patient care [60]. It seems as if the evaluation of earlier initiatives [52] is relevant here as well:

“[…] the effectiveness of government policy is rather limited because of counter strategies of medical specialists. Led by the self-employed, medical specialists have opted in favour of a strategy of collective organisation in hospitals. This strategy is taking the medical specialists and the hospital in a different direction from that envisaged by the government. […] The way in which the integration has evolved might equally well be designated as ‘separation’ rather than ‘integration’ ([52], p.137).

Overall, the organizational and legal attempts for integration over the past decades do not yet fit well with the ideas and interests of medical doctors in the Dutch hospital system. A recent evaluation of the new system of bundled payment since 2015 [60] shows that medical doctors in the boards of Medical Specialist Companies (MSBs) often perceive more influence on improving the quality of patient care; but express that in general, medical specialists seem less engaged: “the involvement of medical specialists can be improved and their attendance at MSB-meetings is often limited.” ([60] p.5). A large majority of the hospital boards in this study is negative about the benefits of the cooperation model. 80 % of the hospital boards in this study perceives the new system as “a costly and time-consuming exercise, while the changes in daily practice have little or no positive impact on patient care” ([60] p.5). Engagement of medical doctors in leadership roles for and beyond direct patient care may require additional initiatives that are closer to their professional values and interests, to let them become engaged in collaborative efforts for more quality and safety and create better cost containment. Governmental and organizational policies with an overly focus on top down performance indicators and competition seem to turn out counterproductive.

## Discussion: lessons from the two healthcare systems

Medical professions have been historically influential in shaping the healthcare systems and the delivery of care [52]. Of course, the degree of autonomy and the influence exerted by the medical profession may vary across various systems and jurisdictions, but it is widely recognized now that medical leadership and engagement in roles that go beyond individual doctor excellence is an important asset for the performance and the future of healthcare system [61, 62]. Most health care systems, in a more or less explicit way, address through reforms or policies the challenge of engaging medical doctors and developing their leadership for health systems improvement [63].

In this paper, based on two jurisdictions, we have analysed the strategies used to mobilize and convert the expertise, ability to influence and legitimacy of the medical profession in an asset to transform and improve health systems. We depart from works in the field of hybridity and medical managers by taking a system approach to the emergence of these roles and attempt to elucidate some of the policy challenges and opportunities to achieve broader engagement of medical doctors in health system reforms and improvement. One of the fundamental policy challenges with regard to the role of the medical profession in the evolution of contemporary healthcare systems is the capacity to create an intermediate space in these systems [64] to mediate and accommodate potentially conflicting forces or pressures to develop transformative and improvement capacities. This implies that governments and healthcare organizations have to search for strategies to locate clinical/medical practice at a more collective level and also to install medical leadership and engagement in so-called less structured systems like networks [65].

To address these challenges faced by mature healthcare systems [39], governments have promulgated policy changes that appear both in Canada and the Netherlands as a combination of top-down policy guidance with bottom-up and joint initiatives between medical bodies and governments. Health systems in Canadian provinces are structurally more centralized than the Dutch healthcare system and less players are involved in their regulation. For health systems in Canada, the main challenge with regard to medical engagement and leadership is to promote collaborative and large-scale improvement initiatives that can provide an arena that is sufficiently distinct and attractive to mobilize the medical profession, and to escape from the constraints and somewhat conflicting tone of the labour regime and the fundamental social contract between the State and the profession. While joint work carries a more voluntary tone, we observed that any serious and enduring medical engagement and leadership development strategy will require some innovative policy for physician compensation and performance management, to take into account the time that physicians are dedicated to extra-clinical roles and to move from local initiatives to broader changes in the system. In addition, the capacity to compensate physicians for leadership development activities, such as training, appears important. Such an innovative policy coupled with capacity development is required to transcend current structural limits of the system.

In the Dutch case, government policies have evolved toward a more market-like model that on the one hand seems to confirm the desire of the medical profession for maintaining a high degree of autonomy, but on the other restricts this autonomy due to limitations set by private health insurance companies and financial policy conditions. One of the challenge of the Dutch system, somewhat similar to the Canadian case, is to channel the variety of initiatives within a coherent and global regulatory framework. As observed, a majority of physicians choose for a model of practice (the Medical Specialist Company) that affirms their status of quasi-autonomous entrepreneurs in the system. As in Canada, there is a constant tension between the desire to nurture professional entrepreneurship (and autonomy) in the system and the need to better connect medical practice to broader organizational and system goals. In the Dutch case, with a very different policy background and a much more plural healthcare system, the situation of the medical profession in terms of autonomy and ability to protect itself from engagement and leadership in extra clinical roles appears basically similar to the Canadian cases.

The challenge for the health system in the Netherlands is therewith also somewhat similar to the one found in the Canadian cases, to promote collaborative and large-scale initiatives that are able to mobilize the medical profession for healthcare improvements beyond a single focus on efficiency and cost containment. For both jurisdictions, the development of medical leadership for health system improvement in various capacities (formal senior executive positions, leaders of improvement initiatives etcetera) seems a pre-requisite to support the wide engagement of the profession in improving the system [62]. However, this is not easy to achieve. An empirical study of medical doctors by Kurunmäki [19] shows that in the health system in Finland considerations for cost management are incorporated in medical practice, blending clinical expertise with accounting principles. The expansion of a process of hybridization to generate large-scale quality improvement appears a persisting challenge, despite earlier initiatives in that direction in the two systems that we analyzed.

Overall, based on a comparison of the two jurisdictions, Canada and the Netherlands, it appears that broad health system’s differences leave open the question how the autonomy and expertise of the medical profession can become a driver of large-scale improvement. In both cases, specific initiatives at the level of the healthcare systems have to be developed to support the involvement of medical doctors in health system improvement. Laissez-faire in this regard will probably provide only small scale and local improvement initiatives in which the role of the medical profession in terms of leadership and engagement is underdeveloped. The development of broader clinical engagement and leadership for health system improvement requires deliberate policy initiatives that can engage the medical profession and governments in some form of joint or collaborative policy-making [66]. Regulatory approaches without such engagement and medical leadership appear insufficient for real healthcare improvement.

## Conclusion

Our paper dealt with the relation between the health system context and the opportunities and constraints for medical doctors to engage in health system improvement, and provide leadership beyond their traditional, though still extremely relevant and demanding, role of care for patients. In this article, we have used illustrations of policy initiatives in two jurisdictions that aimed to influence the role of the medical profession in achieving broad health system improvement goals. While individual clinicians can be fully motivated and engaged in delivering the best care, they seem to depend on a supportive environment to achieve high standards of practice and to use resources wisely [3]. The medical profession plays a key role in developing such environment. The status of the medical profession makes them a unique case in the healthcare system when it is time to foster a more collective view on professional practice. The embeddedness of the medical profession within a highly institutionalized context (labour regimes, tradition of autonomy and arms-length relationship with government and management) obliges policy-makers and the professionals to elaborate strategies and initiatives to compensate the limitations imposed by current institutional rules and discipline [67].

We suggest through this analysis that the use of regulatory constraints is not sufficient to create a durable momentum within the medical profession in order to transcend current limitations. Joint and collaborative policy initiatives that pay serious attention to capacity development and policy coherence can be a promising venue. Capacity development and collaborative work around improvement were discussed in the context of the Canadian cases as an alternative approach to support the involvement of medical doctors in broader organizational and health system goals. In this analysis, strong policy-drive and incentives reveal to be essential components to stimulate interests in joint work at a sufficient scale. In the Dutch case, the use of financial incentives to let the executive board and the medical doctors in hospitals agree on common goals has been predominant. Both countries can learn from these different approaches to better align improvement in capacity development with a proper payment structure. Different institutional contexts, such as a more unitary system like the one found in Canadian provinces and territories and a more pluralist one like the Netherlands, have different policy experiences but seem to face similar policy challenges. Achieving alignment between soft (trust, collaboration) and hard (financial incentives) levers may require facilitative conditions at the level of the health system, like clarity and stability of broad policy orientations and openness to local experimentation [68]. Such policy work will acknowledge the limits of initiatives that overinvest in structural arrangements to engage the medical profession (including financial incentives) and develop its leadership.

## References

[1] Tuohy CH (1999). Dynamics of a changing health sphere: the United States, Britain, and Canada. Health Aff.

[2] Waring J, Currie G (2009). Managing Expert Knowledge: Organizational Challenges and Managerial Futures for the UK Medical Profession. Organ Stud.

[3] Bohmer RMJ (2011). The four habits of high-value Health Care Organizations. N Engl J Med.

[4] Baker GR, Denis J-L (2011). Medical leadership in health care systems: from professional authority to organizational leadership. Pub Money and Management.

[5] Baker GR, MacIntosh-Murray A, Porcellato C (2008). High performing health care systems: delivering quality by design.

[6] Daly J, Jackson D, Mannix J, Davidson PM, Hutchinson M (2014). The importance of clinical leadership in the hospital setting. J Healthc Leadersh.

[7] Pronovost PJ, Miller MR, Wachter RM, Meyer GS (2009). Physician leadership in quality. Acad Med.

[8] Botje D, Plochg T, Klazinga NS, Wagner C (2014). Clinical governance in Dutch hospitals. Clin Governance: An International J.

[9] Denis J-L, Van Gestel N, Kuhlmann E, Blank RH, Lynn Bourgeault I, Wendt C (2015). Leadership and innovation in healthcare governance. The Palgrave International Handbook of Healthcare Policy and Governance.

[10] Spurgeon P, Barwell F, Mazelan P (2008). Developing a medical engagement scale (MES). Int J Cli Leaders.

[11] Ham C, Clark J, Spurgeon P, Dickinson H, Armit K (2011). Doctors who become chief executives in the NHS: from keen amateurs to skilled professionals. J R Soc Med.

[12] McGivern G, Currie G, Ferlie E, Fitzgerald L, Waring J (2015). Hybrid manager-professionals’ identity work: the maintenance and hybridization of medical professionalism in managerial contexts. Public Adm.

[13] Noordegraaf M (2011). Risky business: how professionals and professional fields (must) deal with organizational issues. Organ Stud.

[14] Freidson E (1984). The changing nature of professional control. Annu Rev Sociol.

[15] Waring J (2015). Restratification, hybridity and professional elites: questions of power, identity and relational contingency at the points of ‘professional-organisational intersection’. Sociol Compass.

[16] Nadeem E, Olin SS, Hill LC, Hoagwood KE, Horwitz SM (2013). Understanding the components of quality improvement collaboratives: a systematic literature review. Milbank Q.

[17] Snell AJ, Briscoe D, Dickson G (2011). From the inside out: the engagement of physicians as leaders in health care settings. Qual Health Res.

[18] Schott C, Van Kleef D, Noordegraaf M. Confused Professionals?: capacities to cope with pressures on professional work. Public Management Review 2015; doi:10.1080/14719037.2015.1016094.

[19] Kurunmäki L (2004). A hybrid profession: the acquisition of management accounting knowledge by medical professionals. Account Organ Soc.

[20] Parsons T (1939). The professions and social structure. Soc Forces.

[21] Slater B (2001). The new politics of medical regulation. Soc Sci Med.

[22] Freidson E (1988). Profession of medicine: a study of the sociology of applied knowledge.

[23] Abbott A (2014). The system of professions: an essay on the division of expert labor.

[24] Alexander JA, Waters TM, Burns LR, Shortell SM, Gillies RR, Budetti PP, Zuckerman HS (2001). The ties that bind: interorganizational linkages and physician-system alignment. Med Care.

[25] Shortell SM, Gillies RG, Anderson DA, Morgan Erickson K, Mitchell JB (2000). Remaking health care in America: the evolution of organized delivery systems.

[26] Burns LR, Muller RW (2008). Hospital-physician collaboration. Milbank Q.

[27] Kitchener M, Coronna CA, Shortell SM. From the doctor’s workshop to the iron cage? Evolving modes of physician control in US health systems. Social science and medicine. 2005;60:1311-1322. 10.1016/j.socscimed.2004.07.008 15626526

[28] McKinlay J, Arches J (1985). Toward the proletarianization of physicians. Int J Health Serv.

[29] Starr P (1992). The social transformation of American medicine.

[30] Adler PS, Kwon SW, Heckscher C (2008). Perspective-Professional work: the emergence of collaborative community. Organ Sci.

[31] Exworthy M, Wilkinson EK, McColl A, Moore M, Roderick P, Smith H, Gabbay J (2003). The role of performance indicators in changing the autonomy of the general practice profession in the UK. Soc Sci Med.

[32] Muzio D, Kirkpatrick I, Kipping M (2011). Professions, organizations and the state: applying the sociology of the professions to the case of management consultancy. Curr Sociol.

[33] Parsons W (2004). Not just steering but weaving: relevant knowledge and the craft of building policy capacity and coherence. Aust J Pub Adm.

[34] Denis J-L, Ferlie E, Van Gestel N (2015). Understanding hybridity in public organizations. Public Adm.

[35] Maynard A (2013). Health care rationing: doing it better in public and private health care systems. J Health Polit Policy Law.

[36] Bate P, Mendel P, Robert G (2008). Organizing for quality: the improvement journeys of leading hospitals in Europe and the United States.

[37] Marmor T, Wendt C (2012). Conceptual frameworks for comparing healthcare politics and policy. Health Policy.

[38] Böhm K, Schmid A, Götze R, Landwehr C, Rothgang H (2013). Five types of OECD healthcare systems: empirical results of a deductive classification. Health Policy.

[39] Lazar H, Lavis J, Forest PG, Church J (2013). Paradigm freeze: why it is so hard to reform health-care policy in Canada. Montreal and Kingston: McGill-Queen’s Press for the Institute of Intergovernmental Relations.

[40] Tuohy CH (2012). Reform and the politics of hybridization in mature health care states. J Health Polit Policy Law.

[41] Canadian Health Act. http://laws-lois.justice.gc.ca/eng/acts/c-6/page-2.html#docCont. Accessed Jan 2016.

[42] OECD. Health at a Glance 2013: OECD Indicators, OECD Publishing. 2013. http://dx.doi.org/10.1787/health_glance-2013-en. Accessed Jan 2016.

[43] Mossialos E, Wenzl M, Osborn R, Anderson C (2014). International profiles of health care systems.

[44] Joumard I, André C, Nicq C. Health Care Systems: Efficiency and Institutions. OECD Economics Department Working Papers. No. 769. OECD.44. OECD (2014) OECD Health Statistics 2014. How does Canada compare? Available at: http://www.oecd.org/els/health-systems/Briefing-Note-CANADA-2014.pdf 2010. Accessed Jan 2016.

[45] Horton R (2015). Offline: Medical leadership - from inspection to inspiration. Lancet.

[46] Shared Care (British Columbia) – Available at: http://www.sharedcarebc.ca/. Accessed Jan 2016.

[47] Doctors of BC (2014). Policy Papers: Partnering with Physicians. https://www.doctorsofbc.ca/sites/default/files/policy-partneringwithphysicians-january212014.pdf. Accessed 25 Apr 2016.

[48] Health Quality Council (2012). Accelerating Excellence in Saskatchewan, Government of Saskarchewan.

[49] Health Force Ontario. Government of Ontario. 2015. Available at: http://www.healthforceontario.ca/en/Home/Physicians/Training_%7C_Practising_Outside_Ontario/Physician_Roles/Family_Practice_Models. Accessed Jan 2016.

[50] Gouvernement du Québec and FMOQ. Entente de principe intervenue entre la FMOQ et le MSSS afin d’accroître et d’améliorer l’accessibilité aux services médicaux de première ligne, 11 pages. 2015. Available at : http://www.fmoq.org/Lists/FMOQDocumentLibrary/fr/Presse/Communiqu%C3%A9s/2015/EntenteFMOQ-MSSS-HorsPL2025-05-2015%20.pdf. Accessed Jan 2016.

[51] Kok L, Houkes A, Tempelman C (2010). De relatie tussen medisch specialisten en het ziekenhuis.

[52] Scholten GRM, Van der Grinten TED (2002). Integrating medical specialists and hospitals. The growing relevance of collective organisation of medical specialists for Dutch hospital governance. Health Policy.

[53] Brancheorganisaties Zorg (2010). Zorgbrede Governancecode.

[54] Raad voor de Volksgezondheid en Zorg ‘Governance en Kwaliteit van Zorg’, Den Haag, 2009. https://www.raadrvs.nl/publicaties/item/governance-en-kwaliteit-van-zorg/downloads.

[55] Raad voor de Volksgezondheid en Zorg, briefadvies ‘Relatie medisch specialist en ziekenhuis in het licht van kwaliteit van zorg’, 2010. https://www.raadrvs.nl/uploads/docs/Advies_-_relatie_medisch_specialist_en_ziekenhuis_in_het_licht_van_de_kwaliteit_van_zorg.pdf.

[56] Algemene Rekenkamer (The Netherlands Court of Audit). Implementatie Kwaliteitswet Zorginstellingen geëvalueerd. Tweede Kamer, vergaderjaar 2008–2009, 31 961, nrs. 1–2, Den Haag: Sdu, 2009.

[57] Inspectie voor de Gezondheidszorg, de Staat van de Gezondheidszorg (SGZ) 2009 ‘De vrijblijvendheid voorbij. Sturen en toezicht houden op kwaliteit en veiligheid’. Den Haag, 2009. https://www.rijksoverheid.nl/documenten/kamerstukken/2009/11/27/rapport-staat-van-de-gezondheidszorg-2009.

[58] Inspectie voor de Gezondheidszorg, Toezichtkader voor de invulling van bestuurlijke verantwoordelijkheid van zorginstellingen voor kwaliteit en veiligheid, Utrecht, 2011. http://www.igz.nl/Images/2011-04%20Toezichtkader%20bestuurlijke%20verantwoording_tcm294-303158.pdf.

[59] Schraven T (2011). Over governance in de zorg en de medisch specialist als eigenaar van het ziekenhuis. Medisch Specialistische Zorg 2012, chap 15.

[60] Koelewijn W, Houwen L, Hooge E, Slappendel R, Van der Meer N (2016). Op weg naar gezamenlijkheid. Rapportage Quick Scan integrale bekostiging en governance.

[61] Bohmer R. The instrumental value of medical leadership: engaging doctors in improving services, available at: http://www.kingsfund.org.uk/sites/files/kf/instrumental-value-medical-leadership-richard-bohmer-leadership-review2012-paper.pdf. 2012. Accessed Jan 2016.

[62] Spurgeon P, Long P, Clark J, Daly F (2015). Do we need medical leadership or medical engagement?. Leadersh Health Serv.

[63] Spurgeon P, Clark J, Ham C (2011). Medical leadership: from the dark side to centre stage.

[64] Reed M, Buchanan DA, Bryman A (2009). Critical realism: philosophy, method, or philosophy in search of a method. The sage handbook of organizational research methods.

[65] Ferlie E, FitzGerald L, McGivern G, Dopson S, Bennett C (2013). Making wicked problems governable? The case of managed networks in health care.

[66] Oborn E, Barnett M, Dawson S (2013). Distributed leadership in policy formulation: a sociomaterial perspective. Organ Stud.

[67] Zietsma C, Lawrence TB (2010). Institutional work in the transformation of an organizational field: The interplay of boundary work and practice work. Adm Sci Q.

[68] Hunter DJ (2015). Health policy and management: in praise of political science. Inter J Health Policy Manage.

